# Paradox of life after work: A systematic review and meta-analysis on retirement anxiety and life satisfaction

**DOI:** 10.1371/journal.pgph.0003074

**Published:** 2024-04-04

**Authors:** Lawrence Ejike Ugwu, Wojujutari Kenni Ajele, Erhabor Sunday Idemudia

**Affiliations:** Department of Social Science, Faculty of Humanities, North-West University, Mafikeng, South Africa; University of Palermo, ITALY

## Abstract

Retirement is a pivotal life transition that often changes routines, identity, and objectives. With increasing life expectancies and evolving societal norms, examining the interplay between retirement anxiety and life satisfaction is vital. This study delves into this relationship, recognising the complexities of retirement. A systematic review and meta-analysis followed PRISMA guidelines. Research from 2003 to 2023 was sourced from databases like CINAHL, PubMed/Medline, PsycINFO, ERIC, and Google Scholar, focusing on diverse methodologies and outcomes related to retirement registered in Prospero database (CRD42023427949). The quality assessment used an eight-criterion risk of bias scale, and analyses included qualitative and quantitative approaches, such as random-effects meta-analysis and moderator analyses. After reviewing 19 studies with varied geographical and demographic scopes, a mixed relationship between retirement and life satisfaction emerged: 32% of studies reported a positive relationship, 47% were negative, and 21% found no significant correlation. Meta-analysis indicated high heterogeneity and non-significant mean effect size, suggesting no consistent impact of retirement on life satisfaction. Moderator analyses highlighted the influence of measurement tools on outcomes. The findings reveal a complex interplay between retirement anxiety and life satisfaction, stressing the need for holistic retirement policies that encompass mental health, social integration, and adaptability, focusing on cultural sensitivity. Challenges include potential biases in data sources, methodological diversity, the scarcity of longitudinal studies, and difficulties in addressing recent societal shifts, like the COVID-19 pandemic. Variability in measurement tools and possible publication bias may have also influenced results. This study contributes to understanding retirement, emphasising the relationship between retirement anxiety and life satisfaction. It advocates for ongoing, detailed, culturally informed research to grasp retirement’s multifaceted aspects fully.

## Introduction

Retirement, a milestone in an individual’s life, signifies the end of formal employment and the onset of a new phase characterised by increased leisure and personal pursuits [1, 2]. This transition from a structured, work-centric lifestyle to a period marked by exploration and self-discovery can be profound, often introducing new challenges that significantly alter the retirees’ daily routines and sense of purpose [3]. With increasing life expectancies and healthcare advancements, retirement is often considered an extended period of active, fulfilling life [4]. However, this transition is not without its challenges. The psychological adjustment to retirement is multifaceted, involving various elements such as an individual’s passion for work, the availability of tangible and emotional support, and overall emotional well-being [5, 6]. These factors determine the quality of life post-retirement, ideally marked by satisfaction, fulfilment, and retirement anxiety.

As individuals approach this new phase, many experience “retirement anxiety,” a prevalent concern stemming from uncertainties about post-retirement life [7, 8]. This retirement anxiety often arises from a multitude of factors, including concerns over financial stability, loss of professional identity, social isolation, and health-related issues [9, 10]. Such apprehensions can profoundly impact the retirees’ psychological and emotional well-being.

A diverse range of factors influences the quality of life in retirement [11]. Quality of life in retirement can be described as a retiree’s overall well-being, encompassing physical, mental, economic and social health and their perception of their place in life, including their goals, expectations, and concerns.

Health and physical activity are equally crucial in retirees’ quality of life in retirement, with studies by Božić and Zelenović [12] and Salerno et al. [13] demonstrating that good health and regular physical activity are essential to higher life satisfaction and reduced depression in retirees. Kim [14] observed that social engagement is vital, where active social life and community involvement correlate with increased well-being.

Haslam et al. [15] identified psychological factors, including adaptation to the loss of professional identity and finding a new purpose critical for the mental health of retirees. Furthermore, transitioning into retirement contributed to positive satisfaction levels, with a more favourable gradual transition [16]. Post-retirement employment enhances life satisfaction, especially when it aligns with personal interests [17]. These multifaceted aspects underscore the complexity of the retirement experience and its varying impact on individual life satisfaction.

The existing literature reveals diverse perspectives on the retirement experience. Studies have shown that individuals’ expectations about their retirement significantly impact their satisfaction levels during the initial retirement year [18], underscoring the importance of managing and aligning these expectations to ensure a smoother transition.

Literature indicates that pre-retirement circumstances are crucial in determining mental well-being in later life [19]. This finding highlights the need to understand the various dynamics of retirement to ensure better mental health outcomes for retirees. Moreover, the retirement experience is not uniform across individuals. Diverse factors such as the nature of the occupation, educational level, family dynamics, and geographic location necessitate different approaches to cater to retirees’ varying needs and experiences [20].

Recent research also focused on the prospect of post-retirement employment. Studies indicate that staying engaged in employment post-retirement can positively influence life satisfaction, particularly for those with lower pension incomes [21, 22]. This highlights the potential benefits of continued engagement in work-related activities even after retiring.

In our study, “retirement anxiety” refers to the wide range of worries and fears linked to transitioning from work to retirement. It covers concerns from financial stability and loss of identity to adapting to new routines and maintaining social connections, reflecting the complexity of emotional and psychological challenges faced during this life change. By including studies on related concepts, even when not labelled as “retirement anxiety,” our analysis aims to understand how these various aspects of retirement concern affect life satisfaction. This comprehensive view recognises retirement as a crucial phase with diverse effects on well-being, essential for grasping how retirement anticipation and experience influence life contentment.

Despite the growing body of research, discrepancies remain regarding the impact of retirement on life satisfaction and the onset of retirement-related anxieties. The role of tangible and emotional support in shaping retirement satisfaction is another dimension that requires comprehensive exploration [23]. Given these diverse findings and the significance of retirement in an individual’s life, this systematic review and meta-analysis seeks to synthesise the existing literature on retirement anxiety and life satisfaction. The insights gained from this study can guide future researchers, policymakers, and mental health professionals in devising strategies and interventions to enhance the well-being of retirees. Specifically, our objectives were (1) to analyse the relationship between retirement anxiety and life satisfaction in 2003–23 and (2) to analyse other factors (sample characteristics) in the relationship between retirement anxiety and life satisfaction.

## Methods

We conducted a systematic review and meta-analysis of the paradox of life after work to assess the relationship between retirement anxiety and life satisfaction. This meta-analysis included data from 19 studies, and the review method adhered to Quality In Prognosis Studies (QUIPS) [24]. The QUIPS tool is valuable for assessing the risk of bias in observational studies within systematic reviews and meta-analyses. It guides researchers through a structured evaluation of six key domains—study participation, attrition, measurement of prognostic factors, confounding measurement and account, outcome measurement, and analysis and reporting—to ensure a comprehensive appraisal of each% study’s quality and the reliability of its findings [24]. This systematic approach is crucial for identifying potential biases in observational studies, thereby enhancing the validity of conclusions drawn from systematic reviews and meta-analyses focused on prognostic factors.

This systematic review and meta-analysis was conducted in accordance with the Preferred Reporting Items for Systematic Reviews and Meta-Analysis (PRISMA) guidelines [25] (see S1 Checklist) and was registered prospectively on 13/11/2023 on the PROSPERO International Register of Systematic Reviews (Registration no: CRD42023427949) database.

### Search strategy

The systematic review followed a predefined search strategy. Databases like PubMed, PsycINFO, CINAHL, Google Scholar, and Medline were queried for a timeframe of twenty years, so relevant studies published between January 2003 to September 2023 were included. Keyword adopted includes retirement anxiety, retirement stress, retirement distress, post-retirement anxiety, retirement transition, post-career anxiety, life satisfaction, subjective well-being, quality of life, psychological well-being, happiness, life contentment, retirees, post-retirement individuals, elderly, older adults, seniors, and retirees. We combined these keywords using Boolean operators to create effective search strings such as (retirement anxiety OR retirement stress OR retirement distress OR post-retirement anxiety OR retirement transition OR post-career anxiety) AND (life satisfaction OR subjective well-being OR quality of life OR psychological well-being OR happiness OR life contentment) AND (survey OR correlational study OR cross-sectional study OR longitudinal study OR cohort study) AND (retirees OR post-retirement individuals OR elderly OR older adults OR seniors) AND (English Language), and (“retirement anxiety” OR “retirement stress” OR “retirement distress” OR “post-retirement anxiety” OR “retirement transition” OR “post-career anxiety”) AND (“life satisfaction” OR “subjective well-being” OR “quality of life” OR “psychological well-being” OR “happiness” OR life contentment”) AND (“survey OR correlational study” OR “cross-sectional study” OR “longitudinal study” OR “cohort study”) AND (“retirees” OR “post-retirement individuals” OR “elderly” OR “older adults OR seniors”) AND (“English Language”).

### Eligibility criteria

The eligibility criteria for this systematic review encompass studies focused on retirement anxiety and life satisfaction with a particular emphasis on survey and correlational designs. The targeted population includes retirees, post-retirement individuals, elders, older adults, or seniors. The study design criteria include the inclusion of survey studies assessing retirement anxiety and life satisfaction, as well as correlational studies exploring the relationship between these variables. Only peer-reviewed articles published in English within the last 20 years were considered, excluding conference abstracts, posters, editorials, and non-peer-reviewed publications. Outcome measures must explicitly assess both retirement anxiety and life satisfaction, utilising validated instruments or scales. The review aims to include studies conducted in diverse community, healthcare, and workplace settings. These criteria ensure a focused and comprehensive synthesis of survey and correlational studies examining the intersection of retirement anxiety and life satisfaction.

### Data extraction

Data extraction and management for this systematic review involves the development of a standardised extraction form, pilot testing, and training of reviewers to ensure consistency. A structured database organises extracted data, covering study design, participant characteristics (age, gender), methodology, and results (mean, SD, and Pearson correlation values). Quality assurance measures are implemented to verify accuracy, and a version control system is established to track changes. Discrepancies among reviewers are resolved through consensus or involving a third reviewer. The database, maintained in spreadsheet software or specialised tools, is regularly reviewed for completeness and accuracy. Anticipating data synthesis considerations, the final database is made accessible to the research team. This transparent and systematic approach ensures the reliability and reproducibility of the data extraction process, contributing to the robustness of the systematic review (see S1 Data).

### Quality assessment

Quality assessment using the Quality In Prognosis Studies (QUIPS) tool, developed by Hayden et al. in 2013, systematically evaluates key domains in prognosis studies. The six QUIPS domains, including study participation, attrition, prognostic factor measurement, outcome measurement, study confounding, and statistical analysis/reporting, are individually assessed for risk of bias. Relevant information is extracted from included studies, and each domain is independently evaluated, assigning a risk of bias score (low, moderate, high). An overall judgment of the study’s risk of bias is then derived by integrating domain assessments. Discrepancies are resolved through consensus or third-party involvement. This rigorous process ensures a transparent and systematic assessment of the methodological quality of prognosis studies, enhancing the credibility and reliability of the systematic review findings. The online Cochrane Review software Robvis designed risk-of-bias plots [26] (see S1 Fig).

### Data analysis

The systematic review’s data analysis on retirement anxiety and life satisfaction in survey and correlational studies utilized Comprehensive Meta-Analysis (CMA) software, ensuring a robust examination. Comprehensive Meta-Analysis (CMA) software is used for conducting meta-analyses and systematic reviews, offering extensive features to compute effect-size estimates, assess heterogeneity, and perform sensitivity analyses with ease-of-use and high-resolution graphical outputs for researchers [27, 28].

Employing meta-analysis statistical methods, we synthesized findings across studies by calculating correlation effect sizes through random-effects models, considering heterogeneity. Exploration of heterogeneity involved the I^2^ statistic and subgroup analyses. CMA systematically organized and inputted data from diverse studies, maintaining a comprehensive approach. The software’s statistical capabilities, including Fisher R-to-Z transformation for correlation coefficients, facilitated precise effect size calculations, offering a quantitative measure of the relationship. Heterogeneity assessments utilized the restricted maximum-likelihood estimator (REML) method, contributing to a nuanced understanding of variability. CMA’s features extended to addressing publication bias and conducting sensitivity analyses, enhancing result reliability. The systematic review’s application of CMA ensured a standardized, rigorous, and advanced analysis, enhancing the validity of synthesized findings on the intricate interplay between retirement anxiety and life satisfaction in diverse contexts.

### Study selection, data extraction and quality appraisal

Two of this study authors, L.E.U. and W.K.A, independently assessed the identified studies for their relevance in a two-phase process. Initially, they screened studies based on titles and abstracts. Subsequently, they reviewed the full texts for further evaluation. During both stages, disagreements were resolved through mutual agreement and consulting a third senior author, E.S.I. Under E.S.I.’s supervision, the same two authors independently extracted data using a specially designed spreadsheet. This spreadsheet was initially tested on ten randomly chosen papers and adjusted as needed. The data extraction process included gathering comprehensive reference details, the country where the study was conducted, study design, population details, sample size, specifics of the exposure, outcomes of interest, tools used for assessing retirement anxiety and life satisfaction, and quantitative findings such as mean ages (MA) and standard deviations (SD).

### Data pooling and meta-analysis

In the study, we conducted a descriptive analysis to summarise the characteristics of the included studies using ranges and averages. Given the anticipated variability in pre-defined outcomes across studies, such as differences in study design and populations, we used random-effects meta-analyses. This approach allows us to estimate the relationship between retirement anxiety and life satisfaction, accommodating the expected diversity in study results instead of a fixed-effects model that assumes a singular true effect [29]. The random-effects model is particularly suitable in scenarios of high or anticipated heterogeneity.

Our analysis also incorporated studies that provided correlation coefficients and their total sample sizes or mean differences with sample sizes and corresponding correlations, as detailed in Table 1. We used the I^2^ statistic to evaluate heterogeneity and visually examined funnel plots. Publication bias was assessed through visual inspection of funnel plots [30] and by applying the Begg and Mazumdar [26] and Egger et al. [31] tests. All meta-analyses were performed using Comprehensive Meta Analysis Version 4 software.

**Table 1:** Summary of studies include in the meta-analysis.

Author (Date)	Title	Sample size	Geographical setting	Participants	Gender (F%)	Design	validated tool for LS	validated tool for retirement	Age range	Mean_a_(SD)	Measure	Adjustment	Findings
Ugwu et al. (2019)	Pre-retirement anxiety: Development and validation of a measurement instrument in a Nigerian sample	216	Nigeria	civil servant	54.63	Cross-sectional	Yes	Yes	58–62	59.1 (3.48)	LS, RAS	age, gender	Negative relationship
Bozoglan (2015)	Spousal intrusion as a predictor of wives’ marital satisfaction in their spouses’ retirement.	151	Turkey	retirees	100	Cross-sectional	Yes	Yes	unspecified	55.7 (8.70)	SRSI, DAS	income, educational level, length of retirement, number of weekly activities, spousal intrusion	Negative relationship
Hyde et al. (2004)	The effects of pre-retirement factors and retirement route on circumstances in retirement: Findings from the Whitehall II study	3402	United Kingdom	civil servant	32.33	longitudinal	Yes	No	35–55	59.2 (5.10)	GHQ, ATRQ	Marital status, financial security, general health, household assets, sex, age, retirement pattern	Positive relationship
Dingemans & Henkens (2014)	Involuntary retirement, bridge employment, and satisfaction with life: A longitudinal investigation	1248	Netherlands	retirees	25	longitudinal (10 years period)	Yes	No	50–64	54	SWLS, VRD	age, gender, health status, perceived pension shortfall, partner status, children, job satisfaction, supervisory position, occupational level, time in retirement, bridge job in the past	Negative relationship
Taylor et al. (2008)	The effects of retirement expectations and social support on post-retirement adjustment: A longitudinal analysis	37	US	retirees	8	longitudinal	Yes	Yes	unspecified	60.9 (4.14)	SWR, LS	gender, age, social support, expectations, social satisfaction	Positive relationship
Houlfort et al. (2015)	The role of passion for work and need satisfaction in psychological adjustment to retirement	103	Canada	Quebec association of elderly retirees	unspecified	retrospective cross-sectional	Yes	Yes	unspecified	61.8	BPNS, PAR	Passion for work, age of retirement	Positive relationship
Earl et al. (2015)	A matter of time: why some people plan for retirement and others do not	367	Australia	National Seniors Australia members	46	longitudinal	Yes	Yes	45 and above	65.5 (5.9)	RA, LS	gender, age, years retired, time perspective, retirement planning	Positive relationship
Hershey & Henkens (2013)	Impact of different types of retirement transitions on perceived satisfaction with life	1388	Netherlands	The Netherlands Interdisciplinary Demographic Institute (NIDI)	unspecified	longitudinal (6years period)	Yes	No	50 and above	54.1 (2.89)	SWLS	Age, gender, marital status,self-rated health, years of education, wealth	Negative relationship
Bonsanga & Klein (2012)	Retirement and subjective well-being	12011	Netherlands	retirees	unspecified	longitudinal (6years period)	No	No	50–70	59.2 (0.14)	RE, LS	Age, gender, maritalstatus, education, stress, mental health, coping, depression, smoking and alcohol consumption	Negative relationship
Enache (2017)	Psychological and social effects of aging	60	Romanian	retirees	unspecified	experimental	Yes	Yes	58–70	Unspecified	Cattel, QOL	coping strategies	Negative relationship
Adawi et al. (2023)	Effect of retirement on life satisfaction in canada: Evidence from the 2008–2009 Canadian Community health survey- Health aging	30865	Canada	retirees	64.18	Cross-sectional	Yes	No	55–85	67.60 (8.60)	LS, OR	Age, gender, race, migration status, marital status, educational level, physical health, household size, household income, province	Positive relationship
Hussain & Jan (2022)	Adjustment to retirement: effects on lifestyle across time among retirees	1006	Pakistan	retirees	9.64	Cross-sectional	No	Yes	60–80	Unspecified	LS, RADJ	age, gender, well-being	Negative relationship
Hansson et al. (2019)	Beyond health and economy: Resource interactions in retirement adjustment	1924	Sweden	retirees	unspecified	longitudinal	yes	yes	60–66	62.02 (1.65)	LS, BFR	age, gende, educational level, self-esteem, social support, physical health, cognitive ability, autonomy	Not Significant
Tambellini (2023)	Exploring the relationship between working history, retirement transition and women’s life satisfaction	2877	Italy	retirees	100	longitudinal	Yes	No	unspecified	60.1 (0.10)	LS, RT	age, educational level, marital status, number of children,self-perceived health	Not Significant
Carr et al. (2018)	Postretirement life satisfaction and financial vulnerability: the moderating role of control	344	US	retirees	unspecified	longitudinal	Yes	No	51–87	61.43 (5.01)	LBQ	marital status, volunteering job, spouse disability, financial control, age	Not Significant
Cheng & Yan (2021)	Sociodemographic, health-related, and social predictors of subjective well-being among Chinese oldest-old: a national community-based cohort study	49069	China	retirees	36	longitudinal	yes	Yes	80 and above	Unspecified	SWB	age, gender, occupation, marital status,physical health,living alone, leisure activities, caregiver	Negative relationship
Spiegel & Shultz (2003)	The influence of preretirement planning and transferability of skills on naval officers retirement satisfaction and adjustment	672	US	retirees	0.30	longitudinal	Yes	Yes	38–59	45.30 (2.80)	OFQ, RNLS	officer specialty, gender, marital status, age, employment status, income, education, grade ar retirement	Positive relationship
Henning et al. (2022)	Retirement adjustment in Germany from 1996 to 2014	2079	Germany	retirees	unspecified	longitudinal	Yes	Yes	60–65	Unspecified	RS, RA	AGE, age at retirement, education, time between retirement, gender, financial and physical resources	Negative relationship
Hansson et al. (2020)	Disentangling the mechanisms of retirement adjustment: Determinants and consequences of subjective well-being	497	Sweden	retirees	10.66	longitudinal	Yes	Yes	60–66	63.31 (1.52)	LS,TRC	AGE, Gender, self-esteem, social support, financial satisfaction	Negative relationship

Note: M_a_ =Mean age, SD = Standard Deviation, RAS = Retirement Anxiety Scale, BPNS = basic Psychological Need satisfaction, PAR = Psychological Adjustment to Retirement, RA = Retirement Adjustment, LS = Life Satisfaction, RE = Retirement Expectations, LS = Life Satisfaction Index, SWLS = Subjective Well-being scale, ORS = Overall Retirement Satisfaction, GH = General Health, MCW = Motivation to Continue Work, SRSI = Scale of Retired Spousal Intrusion, DAS = Dyadic Adjustment Scale, GHQ = General Health Questionnaire, ATRQ = Attitude to retirement Questionnaire, VRD = Voluntariness of the Retirement Decision; RNLS = Retirement from Navy Life Survey; BFR = Basic Financial Resources; RADJ = Retirement Adjustment; RT = Retirement Transition; LBQ = Leave Behind Questionnaire; OFR = Officer Career Questionnaire; RS = Retirement Satisfaction; TRC = Total Resource Capability

## Results

### Characteristics of included studies

**Fig 1:**
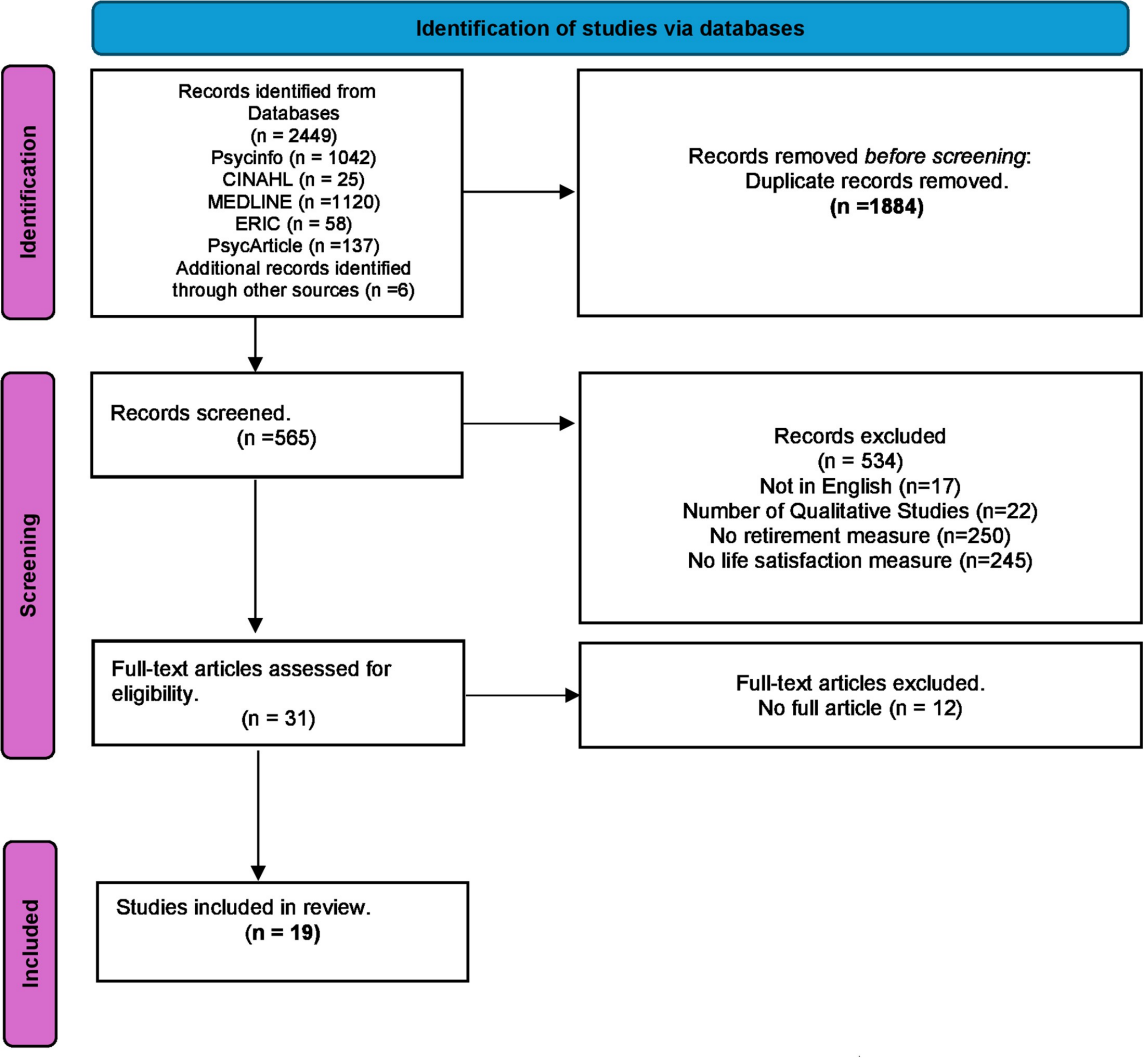
PRISMA diagram of the search and selection process.

We identified 2449 studies by searching the selected databases and listing references of relevant articles. After removing duplicates, 540 records were retrieved. Papers were screened and selected, as illustrated in Fig 1, resulting in 19 papers meeting our inclusion [21, 32–49] papers published between 2003 and 2023. The studies encompass a diverse geographical scope, covering five continents: Africa, America, Australia, Europe, and Asia. The sample sizes of these studies varied significantly, with the smallest being 37 participants [35] and the largest involving 49,069 participants [46], reflecting a broad spectrum of populations.

The studies exhibited a mix of design methodologies. Notably, six studies were cross-sectional, providing snapshots of retirement effects simultaneously. The remaining longitudinal studies tracked changes and trends from 6 to 10 years.

Regarding demographic diversity, the participant pools varied widely in age, with most studies focusing on populations aged between 35 and 80 years. Gender representation also varied, with some studies, such as Bozoglan [33] and Tambellini [44], having a higher percentage of female participants, both of which focused exclusively on female retirees.

Using validated tools to measure life satisfaction (LS) and retirement-related outcomes was common across studies. However, there was variability in adopting these tools, with some studies utilising unique or specifically developed instruments for their research.

Overall, these studies provide comprehensive insights into the multifaceted aspects of retirement, ranging from psychological and social effects to economic and health-related factors, across a wide array of cultural and geographical contexts.

### Retirement and life satisfaction: Qualitative reporting

Our comprehensive analysis of the studies reveals diverse findings on the relationship between retirement and life satisfaction. Of the 19 studies examined, a notable number (n = 6, 32%) reported a statistically significant positive relationship between retirement and life satisfaction, indicating that retirement increased the satisfaction of life [34–37, 41, 47]. Conversely, a larger percentage (n = 9, 47%) found a negative relationship between retirement and life satisfaction, suggesting a decrease in satisfaction post-retirement [21, 32, 33, 38–40, 42, 43, 46, 49]. The remaining four studies (n = 4, 21%) did not find a significant correlation between retirement and life satisfaction [43–45, 48].

The reported correlations in these studies varied, reflecting the complex relationship between retirement and life satisfaction. The studies predominantly used validated tools to measure both life satisfaction and retirement-related factors, enhancing the reliability of their findings. Furthermore, most studies reported adjusted effect estimates, considering variables such as age, gender, health status, and financial situation.

Several studies offered more nuanced insights by differentiating their analysis based on various criteria such as gender, age, and reasons for retirement. For example, Hyde et al. [34] adjusted their findings based on factors like marital status, financial security, and general health, while Dingemans and Henkens [21] took into account variables such as age, gender, health status, perceived pension shortfall, and partner status. This level of detail in the studies highlights the multifaceted nature of how retirement impacts life satisfaction.

Among all the studies, Bonsanga and Klein [39]) factored in the type of retirement (voluntary and involuntary retirement), indicating the negative effect of involuntary retirement on life satisfaction. While other studies (e.g., [41–44, 47] mentioned in their introduction and literature review the adverse effects of involuntary retirement on life satisfaction.

### Retirement and life satisfaction: Quantitative reporting

The meta-analysis consolidated findings from 19 studies with 108,316 subjects, utilising the Fisher r-to-z transformation for correlation coefficients. A random-effects model was the foundation for the analysis, and the restricted maximum-likelihood estimator served as the method to gauge heterogeneity.

**Fig 2:**
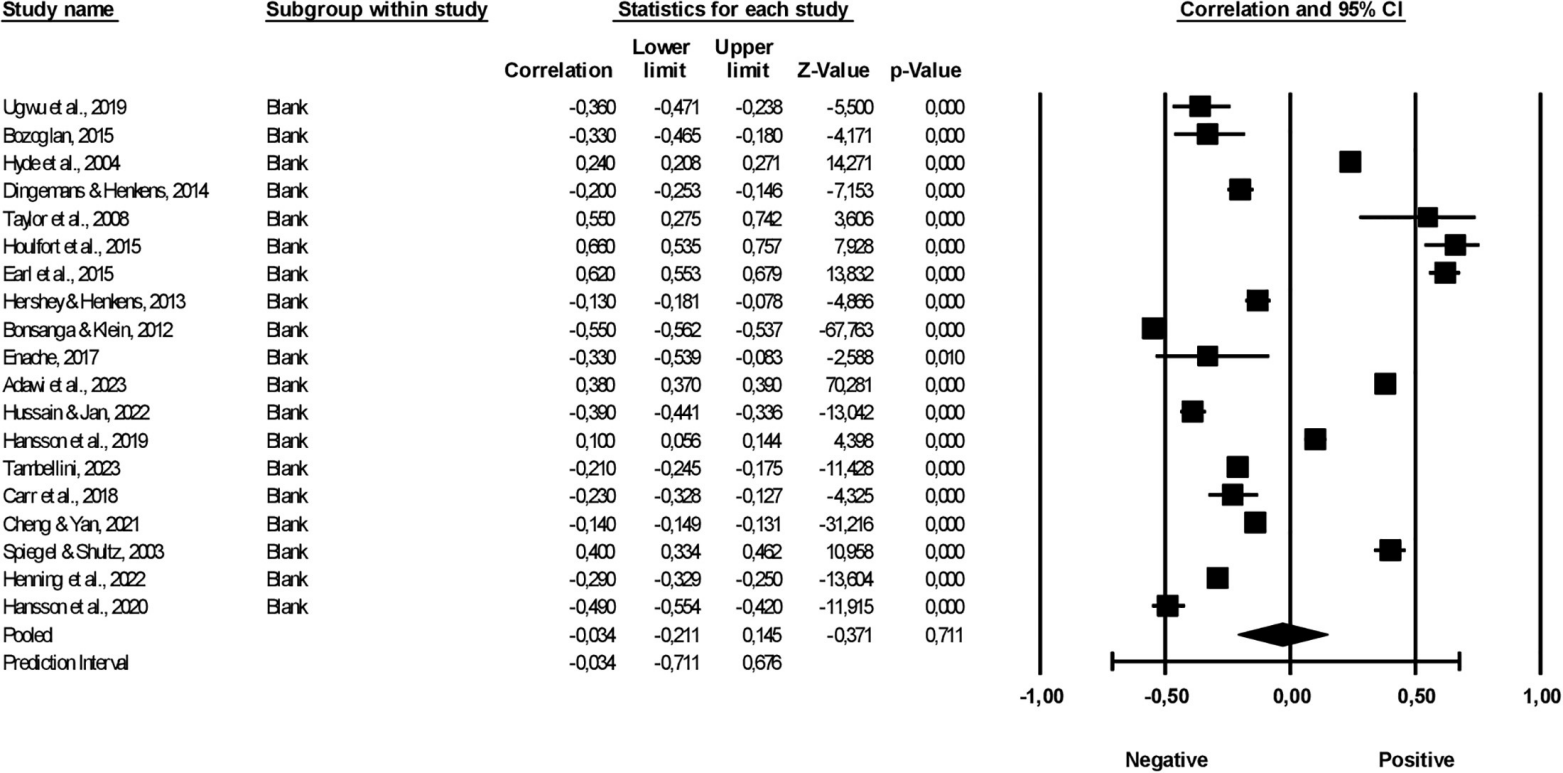
Effect size for the relationship between retirement anxiety and life satisfaction.

When examining the results, the transformed correlation coefficients varied between -0.55 and 0.66. The mean effect size, central to this investigation, was calculated to be -0.034. However, the significance of this effect size is tempered by its wide 95% confidence interval, which spans from -0.211 to 0.145. This broad range indicates high uncertainty around the true mean effect size. Further, the statistical significance of this effect size is questioned as the Z-value stands at -0.371, corresponding to a p-value of 0.711. This value is notably above the conventional alpha criterion of 0.050, leading us to retain the null hypothesis that the mean effect size is zero (see Fig 2).

The analysis also revealed substantial heterogeneity among the studies included. The Q-value evidences this for heterogeneity, a staggering 11,814,055 with 18 degrees of freedom and a p-value of less than 0.0001. Such a result suggests significant variability among the study outcomes. Complementing this, the I-squared statistic stands at 100%, implying that the entirety of the observed variance in effect sizes is attributable to actual differences among the studies rather than mere sampling error.

In terms of variance estimates, tau-squared was determined to be 0.156 in Fisher’s Z units, representing the variance of the true effect sizes. Correspondingly, tau, which denotes the standard deviation of these true effect sizes, was calculated to be 0.395 in Fisher’s Z units.

The prediction interval further contextualises these findings. Assuming a normal distribution of true effects in Fisher’s Z units, we estimated that in 95% of comparable populations, the true effect size would lie between -0.711 and 0.676. This broad interval highlights the diversity of effects that may be encountered in similar studies and populations.

**Fig 3:**
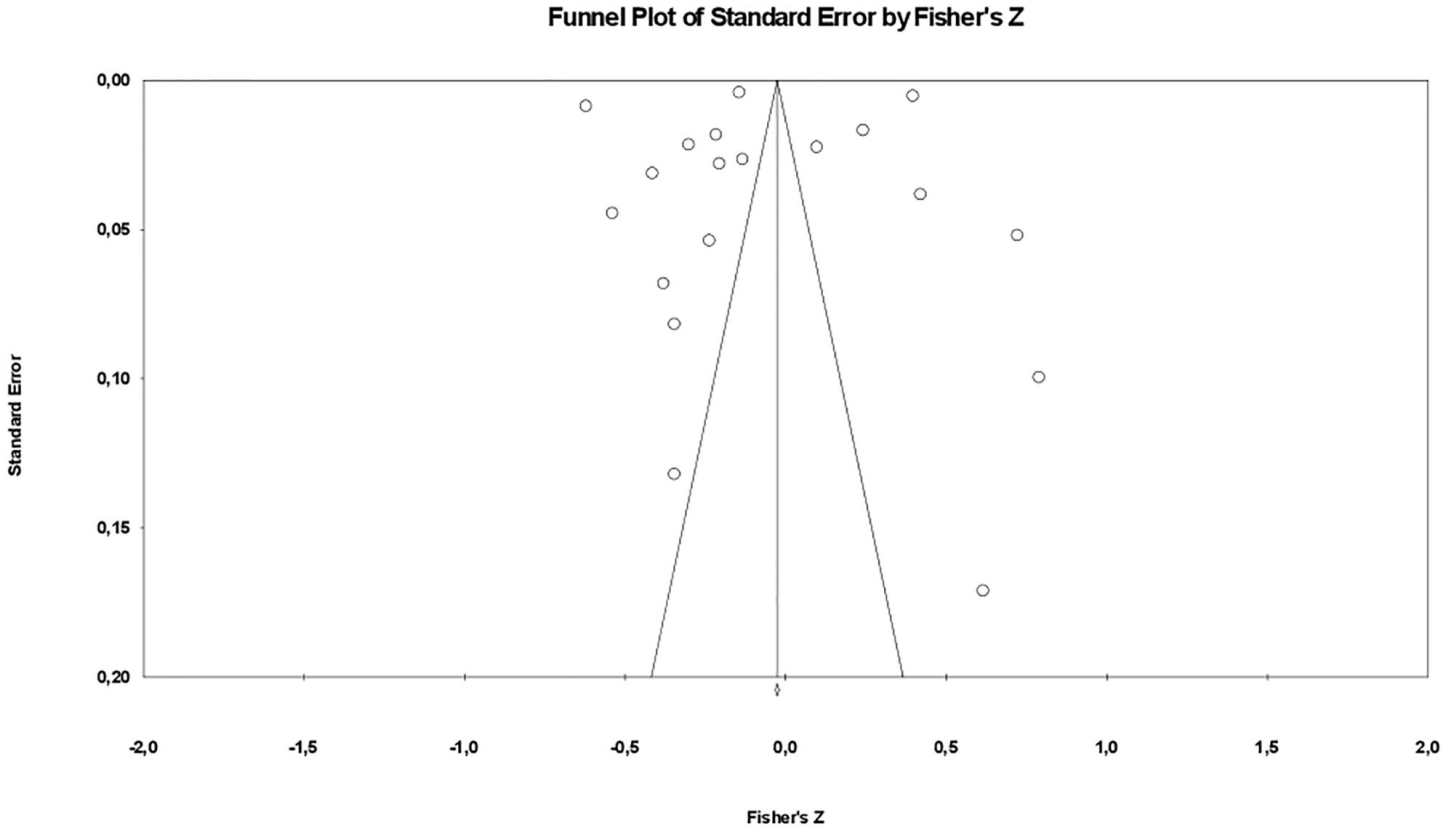
Funnel plot for assessing publication bias.

The funnel plot (see Fig 3) indicated potential asymmetry in the meta-analysis, suggesting publication bias. This was supported by a significant Egger’s regression intercept (p < 0.05) and corroborated by Begg and Mazumdar’s rank correlation (p < 0.1). Despite this, the Classic fail-safe N analysis revealed many studies needed to overturn the effect, indicating the robustness of the findings. Duval and Tweedie’s trim and fill method adjusted for the observed asymmetry, confirming publication bias. Collectively, these analyses enhance the understanding of the potential biases impacting the meta-analysis results.

### Moderator analyses

Moderator Analyses Meta-regressions were only conducted for variables that had four or more studies that provided data on that variable. Two meta-regressions were conducted in total for the comparison analysis. See Table 2 for the statistics for all moderator analyses and each meta-regression. None of the two meta-regressions were statistically significant.

**Table 2:** Comparison meta-regression.

Moderator	No. studies	Q	*p* value	R^2^(%)
Design	19	0.01	0.93	0.33
Place of study	19	16.33	0.0026	0.21

Note. Q is the statistic used to test the significance of the meta-regression. R^2^ is the proportion of variance explained by the moderator. The place of study refers to the continent of each country.

Table 3 shows the results of the subgroup meta-analysis, which explores the impact of different measurement scales on the relationship between retirement anxiety and life satisfaction and research design. This analysis segregates studies using validated and self-developed scales and cross-sectional and longitudinal designs addressing how these methodologies might influence outcomes.

**Table 3:** Subgroup meta-analysis.

Measure	k	r_+_	z-value	LL	UL	Tau	Q	I^2^	p-value
***Independent variable Measures (retirement anxiety)***									
**Validated scale**	6	0.175	11.441	0.145	0.204	0.221	393.456	99.746	.001
**Self-developed scale**	13	-0.135	-43.829	-0.141	-0.129				
**Pooled**		0.020	0.129	-0.279	0.315				
***Dependent variable Measure (life satisfaction)***									
**LS**	8	-0.113	-37.349	-0.119	-0.107	0.173	4047.629	99.901	0.001
**GHQ**	1	0.240	14.271	0.208	0.271				
**SWB**	4	0.022	5.005	0.013	0.031				
**QOL**	1	-0.330	-77.980	-0.338	-0.322				
**Self-developed Scale**	5	0.043	0.325	-0.213	0.294				
**Pooled**		-0.036	-0.444	-0.191	0.121				
***Design***									
**Cross-sectional**	6	0.086	15.503	0.075	0.097	0.054	131.719	99.241	0.001
**Longitudinal**	13	0.010	2.755	0.003	0.017				
**Pooled**		0.048	1.259	-0.027	0.122				

Note: K = Number of studies; r_+_ = Correlation mean; LL = Lower Limit; UL = Upper limit; LS = Life satisfaction scale; GHQ = General Health Questionnaire; SWB = Subjective Well-being; QoL = Quality of life scale

When analysing studies that used validated scales to measure retirement anxiety, a small but significant positive correlation was observed with the outcome variable (mean correlation r+ = 0.175, p = .001). This suggests a modest link between higher levels of retirement anxiety and the corresponding outcomes. However, a high heterogeneity among these studies (I^2^ = 99.746%) suggests that this relationship is inconsistent across different research contexts. This variability implies that factors beyond the primary variables of retirement anxiety and life satisfaction might influence these outcomes, calling for a cautious interpretation of this finding.

Conversely, there is a slight negative correlation in studies employing self-developed scales for measuring retirement anxiety (r_+_ = -0.135). This contrast in the direction of the correlation compared to the validated scales is particularly telling. It highlights how the choice of measurement tool can significantly influence the results, pointing to the potential variability introduced by different scale constructions.

When we pooled the data from all studies, regardless of the scale used, the overall correlation became negligible (r_+_ = 0.020), accompanied by a wide confidence interval. This underlines the complexity and uncertainty surrounding the general effect size, suggesting that a myriad of factors beyond the scope of our analysis might influence the overall association.

Turning to life satisfaction, the results were even more varied, notably across the scales (Life Satisfaction Scale, General Health Questionnaire, Subjective Well-being, Quality of Life, and self-developed scales). This variation underlines the challenging nature of measuring life satisfaction and the varying degrees of associations that emerge depending on the chosen scale. Notably, the Life Satisfaction scale showed a slight negative correlation (r_+_ = -0.113, p = 0.001) but with an extremely high level of heterogeneity (I^2^ = 99.901%), indicating significant variability across studies.

Cross-sectional studies showed a modest positive correlation (r+ = 0.086, p = .001), indicating a weak relationship between retirement anxiety and outcomes at a single point in time. High heterogeneity (I^2^ = 99.241%) within this group indicates variability across research settings, complicating broad conclusions from cross-sectional data. Longitudinal studies revealed an even weaker correlation (r+ = 0.010), suggesting minimal long-term impact of retirement anxiety on life satisfaction. This emphasises the need to consider temporal dimensions in such research.

Pooling data from both study types yielded a small combined effect size (r+ = 0.048), with a wide confidence interval, underscoring the complex relationship between retirement anxiety and life satisfaction. This indicates that effects may depend on various factors, including timing and dynamics of retirement anxiety.

The subgroup meta-analysis paints a complex picture. It reveals that the scale used to measure retirement anxiety and life satisfaction and research designs can profoundly impact research outcomes. The high levels of heterogeneity observed across most categories indicate substantial differences in study methodologies or other unique study aspects. These findings necessitate a cautious approach in interpreting the pooled results and suggest that further exploration, perhaps including additional moderators or other subgroup analyses, could shed more light on the reasons behind this observed heterogeneity.

## Discussion

This systematic review and meta-analysis aimed to elucidate the relationship between retirement anxiety and life satisfaction while examining the influence of various factors on this relationship. Given the increasing life expectancies and the evolving nature of retirement in individuals’ lives, this exploration is critical. The study’s objectives were to analyse the relationship between retirement anxiety and life satisfaction from 2003 to 2023 and to explore the impact of other variables, such as demographic characteristics, on this relationship.

A key finding from the review is the diverse impact of retirement on life satisfaction. Consistent with Hyde et al. [34], we observed instances where retirement positively influenced life satisfaction, likely due to the transition to leisure and personal pursuits. This aligns with the perspective that retirement can be a fulfilment period, as individuals engage in activities they value, echoing findings by Taylor et al. [35]. These studies suggest that the retirement phase, adequate planning, and social support can increase satisfaction.

However, this positive outlook is not universal. The analysis also uncovered studies, such as those by Ugwu et al. [32] and Bozoglan [33], indicating a negative relationship between retirement and life satisfaction. This could be attributed to retirement-related anxieties stemming from financial insecurity, loss of identity, and social isolation, as discussed in Froidevaux et al. [9] and Ujoatuonu et al. [10]. Such factors can profoundly impact retirees’ emotional and psychological well-being, highlighting the need for comprehensive support systems.

The significance of health and physical activity in determining the quality of life during retirement is evident in studies by Božić and Zelenović [12] and Salerno et al. [13]. They propose maintaining good health and engaging in regular physical activities are critical for higher life satisfaction. This echoes Kim’s [14] findings on the importance of social engagement and its correlation with well-being, suggesting that active participation in community life can mitigate feelings of isolation and loss of purpose.

Chen et al. [19] emphasise the role of pre-retirement circumstances in shaping mental well-being post-retirement. This finding indicates that the retirement phase itself does not solely determine the quality of life in retirement but is also significantly influenced by the conditions and preparations leading up to it.

Our review also acknowledges the variability in retirement experiences. Birkett et al.[20] highlighted that factors like occupation, education level, and family dynamics significantly affect retirement experiences, necessitating personalised approaches to support retirees.

Furthermore, the potential benefits of post-retirement employment are evident in studies by Dingemans and Henkens [21] and Markowski et al. [22]. They suggest that continued engagement in work-related activities, especially for those with lower pension incomes, can enhance life satisfaction, aligning with our findings on the multifaceted nature of the retirement experience.

The meta-analysis, utilising a random-effects model and considering heterogeneity across studies, reveals a varied range of effect sizes and substantial heterogeneity. The pooled results indicate a statistically non-significant mean effect size, suggesting no generalisable impact of retirement on life satisfaction. However, the high degree of variability and broad prediction intervals in the results underscore the diverse experiences of retirees and the influence of multiple factors on their life satisfaction.

Moderator analyses, including meta-regressions and subgroup analyses, reveal that the choice of measurement scales for retirement anxiety and life satisfaction significantly impacts outcomes. Studies using validated scales show a small but significant positive correlation between retirement anxiety and outcomes, whereas those with self-developed scales exhibit a slight negative correlation. This disparity underlines the influence of measurement tools on research findings.

### Implication of the study

The research contributes substantially to the existing knowledge on retirement, suggesting a paradigm shift in how retirement is studied and understood. It advocates for an integrative approach that considers the multifactorial nature of retirement anxiety and life satisfaction. This approach must encompass traditional psychological and sociological perspectives while incorporating insights from public health, digital literacy, and global health crises. The study calls for longitudinal research methodologies that capture the evolving nature of retirement experiences in the face of societal changes such as pandemics and technological advancements.

From a policy standpoint, the findings emphasise the urgency for comprehensive and adaptive retirement policies. These policies should extend beyond financial security, addressing broader aspects of retirees’ well-being, including mental health, social integration, and digital inclusion. The study advocates for developing holistic retirement support systems responsive to global health emergencies and technological disruptions. Policies should facilitate lifelong learning and digital literacy among older populations, ensuring their active and meaningful social participation.

Clinically, the study has profound implications for how healthcare providers and mental health professionals approach retirement-related issues. It suggests a need for holistic assessment tools to evaluate retirement’s psychological and social dimensions. Additionally, there is a call for public health initiatives that promote active ageing, focusing on both physical and mental health resilience.

The societal changes underscored by the COVID-19 pandemic and the rise of AI technology provide a critical backdrop for future research. There is a necessity for studies exploring the impact of such global shifts on retirees’ mental health and well-being. Research should also examine the role of digital technologies in enhancing the quality of life for retired populations, assessing both the opportunities and challenges presented by such advancements.

### Limitations of the study

While shedding valuable light on the relationship between retirement anxiety and life satisfaction, the systematic review and meta-analysis have limitations that merit consideration.

Firstly, the scope of data sources in the study poses a significant limitation. While the researchers employed a rigorous search strategy, their reliance on existing literature and databases might have inadvertently overlooked pertinent studies, especially those from underrepresented regions or published in languages other than English. This limitation could affect the universality of the findings, potentially skewing the representation of various cultural and socio-economic backgrounds.

Another notable limitation is the methodological variability among the included studies. The array of methodologies, ranging from cross-sectional to longitudinal designs, provides diverse insights but also introduces inconsistent data quality and interpretability. Notably, the underrepresentation of longitudinal studies, which are critical for understanding the dynamics of retirement over time, might limit the depth of our analysis.

The rapid pace of societal changes, such as the COVID-19 pandemic and advancements in AI technology, presents another challenge. The study may not fully capture the long-term effects of these recent and ongoing developments on retirement experiences, as the impacts are still unfolding, and relevant data is continually emerging.

Additionally, there’s variability in the measurement and operationalisation of retirement anxiety and life satisfaction across the studies we analysed. The use of different instruments, each with sensitivity and specificity, could affect the results’ comparability and consistency, potentially impacting our meta-analysis’s robustness.

Publication bias is also a potential limitation. There is always a risk that studies with significant or positive findings are more likely to be published, leading to an overrepresentation of such studies in our analysis. This bias can provide a skewed understanding of the actual relationship between retirement anxiety and life satisfaction.

Lastly, the interdisciplinary complexity of this research, spanning psychology, gerontology, public health, and technology, poses a challenge in fully integrating and interpreting findings across these diverse fields. This complexity might limit our ability to comprehensively understand the retirement experience in the context of societal and technological changes.

Considering these limitations, the interpretations and conclusions drawn from our study should be approached with a degree of caution. Future research in this field would benefit from a more uniform approach in measurement tools, including longitudinal studies and a broader representation of geographical and demographic groups.

### Future research recommendations

Reflecting upon the identified limitations in our study, we propose a series of recommendations for future research that aim to deepen and broaden the understanding of retirement experiences across diverse cultural and demographic contexts.

Firstly, there is a clear need to standardise measurement tools used in retirement research. Future studies should prioritise developing and using instruments that are universally applicable and sensitive to cultural differences. This would allow for more consistent and reliable comparisons across various studies, enhancing the generalizability of findings.

Emphasising longitudinal research designs is another crucial recommendation. Such studies can track changes in retirement experiences and attitudes over time, offering insights beyond the snapshot provided by cross-sectional studies. This approach would help understand retirement’s evolving nature and long-term impacts on individuals.

Integrating qualitative methods into future research is also essential. Methods like in-depth interviews, focus groups, and ethnographic studies can uncover rich, detailed narratives about the retirement experience. These qualitative insights can reveal the nuanced, subjective dimensions of retirement, providing depth to the findings that quantitative data might not fully capture.

Addressing the cultural biases inherent in many current measurement tools is vital. Future research should focus on developing or adapting new instruments to ensure cultural relevance. This is particularly important for accurately capturing the diverse experiences of retirees from different cultural backgrounds, thereby enhancing the cultural sensitivity and applicability of research findings.

Finally, expanding the scope of research to encompass a broader range of professions, geographic locations, and socio-economic backgrounds, incorporating the type of retirement (voluntary and involuntary) in the study would provide a more holistic view of retirement. Understanding how retirement is experienced across different sectors, cultures, and social strata is crucial for a comprehensive view of this life stage.

Implementing these recommendations in future research endeavours will significantly contribute to a richer, more nuanced understanding of retirement. This approach will enhance academic knowledge and inform more effective policies and interventions to support retirees in various contexts, ultimately leading to improved quality of life and well-being in retirement.

## Conclusion

The systematic review and meta-analysis exploring the relationship between retirement anxiety and life satisfaction from 2003 to 2023 reveals a nuanced landscape. The studies included show mixed results: some suggest retirement boosts life satisfaction, others report a decline, and a few observe no significant change. These varied findings highlight the complex nature of retirement’s impact.

Significant heterogeneity characterises the research findings. This variability mirrors the factors influencing retirement. It underscores the intricate nature of studying this life phase, as evidenced by the meta-analysis’s wide range of effect sizes and confidence intervals.

The choice of measurement tools heavily influences the outcomes of studies on retirement anxiety and life satisfaction. The analysis reveals considerable variation in results based on the scales used, highlighting the critical role of methodology.

Moderator analyses for variables like study design and geographical setting did not show statistically significant impacts, suggesting these factors may not substantially affect the relationship between retirement anxiety and life satisfaction.

The review points to potential publication bias, as the funnel plot analysis indicates. However, the Classic fail-safe N analysis lends some support to the robustness of the findings despite adjustments confirming bias presence.

Subgroup meta-analysis adds complexity, showing that the scale used for measuring retirement anxiety and life satisfaction leads to different outcomes. This highlights the challenges in measuring these constructs and the varied associations based on the scales used.

## Supporting information

S1 Fig Risk-of-bias plots. (TIF)

S1 Checklist PRISMA abstract checklist. (DOCX)

S1 Data Retirement meta-analysis data. (XLSX)
