# Supplementary figures and images for: Paradox of life after work: A systematic review and meta-analysis on retirement anxiety and life satisfaction
Source: PLOS Glob Public Health. 2024 Apr 4;4(4):e0003074. doi: 10.1371/journal.pgph.0003074 (PMC10994353; doi:10.1371/journal.pgph.0003074)

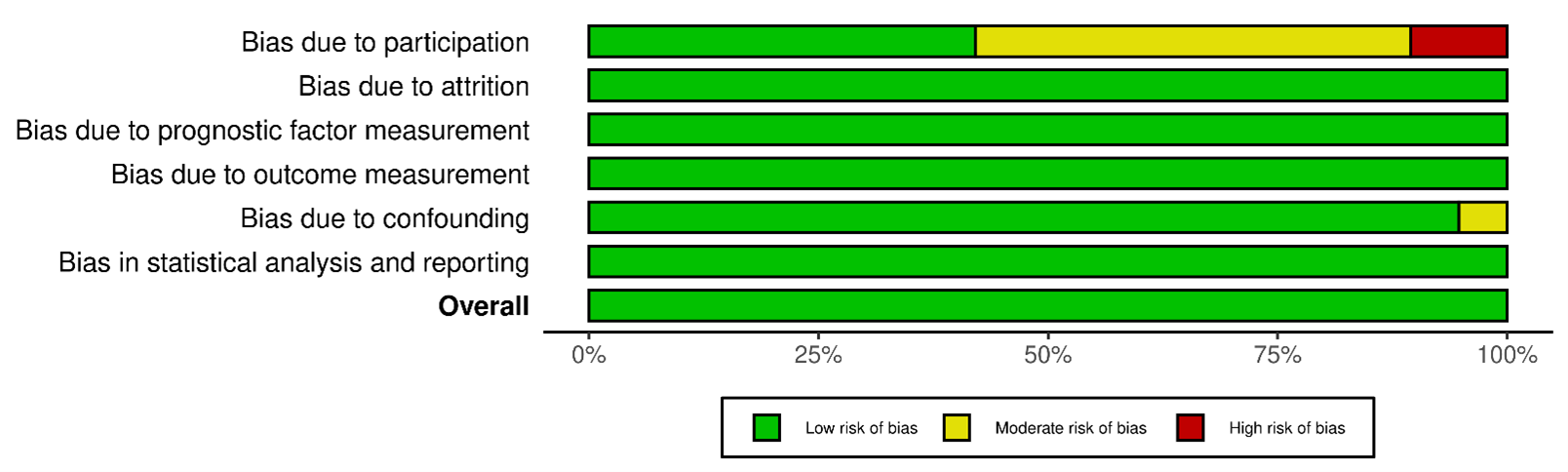

Supplement: S1 Fig — (TIF) [file pgph.0003074.s001.tif]
